# Efficiency of Unitraps in Capturing Corn Earworm Moths, *Helicoverpa zea* (Lepidoptera: Noctuidae), in the Field

**DOI:** 10.3390/insects16050525

**Published:** 2025-05-15

**Authors:** Gabriel P. Hughes, Ring T. Cardé

**Affiliations:** Department of Entomology, University of California Riverside, Riverside, CA 92521, USA; gabrielphughes@gmail.com

**Keywords:** trap efficiency, pheromone, video observations

## Abstract

Pheromone-baited traps are commonly used to monitor and detect moths. Traps and lures are often compared to each other to identify the optimum set up and lure dosage. However, it is also important to understand the efficiency of a trap in capturing the moths that are attracted to it. In the present study, three pheromone-baited traps were placed at the edge of a cornfield to determine the one with the highest capture rate of *Helicoverpa zea* (Lepidoptera: Noctuidae): Scentry Heliothis traps, clear Unitraps, and green Unitraps. Once it was determined that green Unitraps captured more *H. zea*, field observations determined the number of moths captured in the traps compared to the number approaching, i.e., trap efficiency. Green Unitraps had a capture efficiency ranged from 5 to 11%, with an average of 11%. Unitraps, although useful for monitoring existing populations, may not be effective in detecting an invasive incursion. The implications of low capture efficiency in the surveillance of *H. zea* are considered, including possible explanations and next steps to improve monitoring efforts of heliothine moths.

## Simple Summary

Monitoring efforts for moths often rely on pheromone-baited traps to capture males. Trap characteristics, including color and shape, are often compared by determining which design captures the most moths. However, few studies have investigated the number of moths captured compared to the number that approach the trap, i.e., trap efficiency. Three traps were compared for total number of moths captured, and the best trap, the green Unitrap, was then used in field observations to determine its capture efficiency. Moths were videoed at night, and the number approaching the trap was recorded and compared to the number trapped. On average, 11% of moths orienting to the trap were captured. Understanding the capture efficiency of traps will help in interpreting monitoring and surveillance efforts and set the stage for improvements to trap designs.

## 1. Introduction

Pheromone-baited traps are a mainstay in the monitoring and detection of moths. In addition to the wide variety of lure types used to capture these insects, a variety of traps are employed to take advantage of the behavioral patterns and sizes of different species. For helothine moths, the “bucket” trap or universal moth trap (Unitrap) is widely used due to its low cost, the ease with which the trap can be serviced, and its durability in the field [1,2]. Bucket traps consist of a bucket containing a killing agent, usually dichlorvos, with a dry funnel affixed to the top. A lid is situated 3 cm above the funnel, and a small plastic basket suspended from the lid over the funnel entrance holds the lure. The moth flies toward the lure and if it falls through the funnel into the bucket it is killed by the volatile insecticide. Another common archetype is the Hartstack trap [3], which has a pheromone lure at the base of a hollow, metal mesh cone that attracts moths and directs them up to a collection cup situated at the top of the cone. A variation on the Hartstack trap, the Scentry^®^ Heliothis trap (hereafter referred to as “Scentry trap”), uses the same general design but is constructed with a white vinyl mesh that allows the trap to be collapsed for more convenient storage. An understanding of insect behavior can improve trap design [4], and this trap design takes advantage of the behavior of male Heliothine moths to climb up inner surface after landing into the collection receptacle. In some studies, the Hartstack and Scentry traps captured more moths than the Unitrap [2,5,6].

The turbulent structure of the plume emanating from a trap can also modulate moth orientation and trap captures [7]. Even the placement of a lure within a trap can influence capture [8]. Trap color is another important consideration. For spruce budworm (*Choristoneura fumiferana* (Clemens), the color of Pherocon^®^ 1C and 1CP traps (based off the Howell trap) [9] and diamond-shaped Sectar traps did not have a strong effect on capture rates, except that yellow traps were less effective than green, blue, or white traps [10]. However, for moths in the Noctuidae, it appears that darker colors such as forest green which had previously been a common trap color, capture fewer moths than yellow and orange [11,12,13]. Yellow traps also capture more bycatch, such as bees and wasps [14].

Lure, plume structure, trap configuration, and trap color all can combine to affect the number of moths that are captured. Several studies have examined the relative captures between several trap or lure types [6,15], but few have compared the number of moths attracted to or approaching traps, with the number captured, i.e., trap efficiency [16,17]. In one study, a higher proportion of European corn borers *Ostrinia nubilalis* (Hübner) approaching pheromone-baited Scentry traps were subsequently captured (72%) compared with the proportion of approaching moths that were captured in sticky wing traps (4%) [16]. Field observations of the spongy moth, *Lymantria dispar* (L.), found that trapping efficacies of >80% for the standard green Delta survey trap, <15% for the Unitrap [16] and 10% for the green Milk-Carton trap [18]. In surveying for the presence of the spongy moth, approximately 200,000 Delta traps are deployed yearly in the United States [19]. The probability of detection and avoiding a false negative are contingent on distance between traps, lure strength which dictates a trap’s potential drawing distance, and a trap’s efficiency of capture [20].

*Helicoverpa zea* (Boddie) (Lepidoptera: Noctuidae) is an important pest of corn, tomato, cotton and many other crops throughout the Americas. These moths are abundant near cornfields during the summer in southern California, making them an ideal subject for experiments. The purpose of this study was to determine the most suitable trap for *H. zea* and to determine through video observations the trapping efficiency of that trap type. *Helicoverpa zea* has yet to invade Eurasia and this pest is of great concern [21], and so pheromone-baited traps that are highly efficient for detection are desired. Knowledge of trapping efficiency will aid monitoring programs in understanding the limitations of drawing conclusions from trapping data, as well as informing new traps or modifications to existing trap designs.

## 2. Materials and Methods

Field experiments used pheromone lures composed of gray rubber septa (West Pharmaceuticals, Exon, PA, USA) that had been cleaned with dimethyl chloride using soxhlet extraction. Cleaned rubber septa were allowed to dry in a fume hood and were then loaded with 3 mg of a synthetic mixture of *Z*-11 hexadecenal and *Z*-9 hexadecenal at a ratio of 97:3 (Bedoukian Research, Danbury, CT, USA).

To determine the effect of trap type and color on the attraction of *H. zea*, three styles of traps were deployed in the field in Imperial Co., Brawley, CA, USA (32.9333° N, 115.4047° W): a green Universal moth trap (Unitrap), a clear Unitrap (Great Lakes IPM, Westerburg, MI, USA), and a Scentry trap (Scentry Biologicals, Billings, MT, USA) constructed of white, vinyl mesh. The site included three plots of corn each totaling ca. 6 ha. Unitraps were affixed to green metal stakes using wire so that the lure height was 1 m above the ground. The Scentry trap was tied to a 1.5 m-long stake such that the opening at the bottom of the trap was 1 m above the ground. Three replicates, each containing the three trap styles (nine traps in total), were set on the west edge of cornfields >50 m apart, and the distance between traps within each replicate was 10 m. The traplines were on bare soil 10 m from the edge of the cornfield and parallel to that edge. While traps are typically placed perpendicular to the prevailing wind, the wind at the study site was erratic, with no apparent prevailing directionality. The lure was placed in the lure basket of each bucket trap and clipped to the strip of fabric that spans the opening of the base of Scentry traps. Unitraps also received an insecticidal strip (VaporTape II^®^, Hercon Environmental, Emigsville, PA, USA) (active ingredient: 10% Dichlorvos) in the bucket of the trap to kill any moths that entered. Traps were set on 11 May 2018, and were checked weekly through to 2 June 2018. The temperature during this time period averaged 31.1 °C, with a maximum of 40 °C and a minimum of 19.4 °C. Traps were emptied into labeled resealable bags in the field, and the collection buckets reattached. The moths were transported to the lab where they were counted. Each time the traps were checked, the treatments, including the trap to which they were affixed, were moved one position along the line of traps such that each trap occupied each position in the transect once during the experiment. Each trap count comprised a replicate: three transects checked three times.

The second experiment was conducted at the University of California Riverside Agricultural Operations field site (Riverside, CA, USA) (33.9643° N 117.3416° W) from 15 June to 2 July 2019 on a 0.4 hectare plot at least 25 km from other corn plantings. Approximately 9000 corn seeds were planted in late spring (between 25 March and 9 April) and drip tape was used for irrigation. The plots were treated once a week on a rotation of Bt kurstaki, methoxyfenozide, and spinetoram. The temperature during this time period averaged 20 °C, with a maximum of 33.9 °C and a minimum of 16.1 °C. Nightly windspeed began around 10 km/h at 20:00 and decreased gradually to 0 km/h by 03:00 the following morning. One pheromone-baited Unitrap was placed at the periphery of this corn field to monitor population levels to inform the timing of the experiment. A second trap was set up in the cornfield at dusk in preparation for the behavioral observations during the active hours of male *H. zea*. The trap was recorded from dusk until dawn using a video camera (Sony Handycam HDR-CX405; Sony Electronics Inc., San Diego, CA, USA) in Nightshot (infrared) mode with an infrared light attachment. The camera was set up on a tripod so that the viewing angle was perpendicular to the prevailing wind. This allowed for the flight path of male *H. zea* to be viewed as they flew upwind toward the trap. Observations were conducted on five separate nights. The video footage was later viewed to categorize the behaviors of *H. zea* males attracted to the lure. Specifically, three events were recorded: approach, contact, and capture. Approach was defined as the upwind flight of a moth toward the trap, usually involving a decrease in flight speed accompanied by surging movement toward the odor source. This contrasts with “fly-bys”, in which a moth would enter the frame of the video, fly past the trap without slowing, and then leave the frame. Contact was defined as a moth physically touching the trap, which included landing on the trap or bumping into the bucket or the basket containing the lure. Capture was defined as a moth entering the trap and not being observed to escape. These events were sequential such that a moth that was captured also exhibited approach and contact behaviors.

Data for both experiments were analyzed using a Kruskal–Wallis test followed by a Dunn multiple comparisons test using the dplyr package [22] in R version 4.0.3 [23]. In the first experiment, the independent variable was trap type (with three levels: green Unitrap, clear Unitrap, and Scentry trap), and the dependent variable was number of moths captured in each trap each week. Pairwise comparisons were made between transects and between trap dates to test if data could be grouped in a single dataset. In the second experiment, the independent variable was a given behavior (with three levels: approach, contact, and capture), and the independent variable was the number of moths exhibiting each behavior. Even though moths that were captured also exhibited approach, and contact behaviors, only the final behavior was counted for analysis, i.e., captured moths were not also counted as approaching or contacting.

## 3. Results

**Figure 1:**
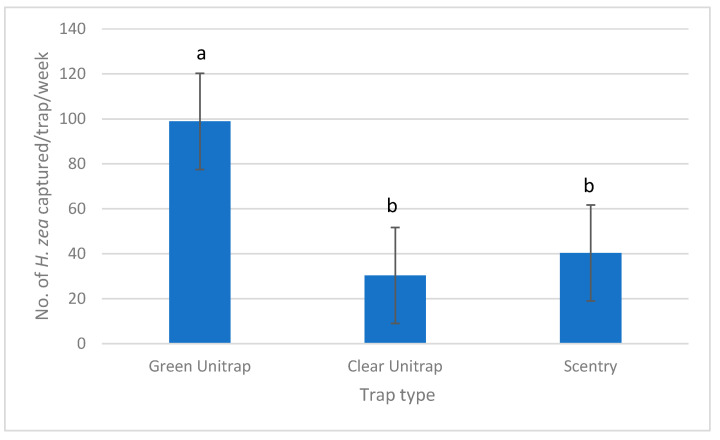
Mean number of *H. zea* captured in three different trap types: green Unitrap (890 total moths), clear Unitrap (273 total moths), and Scentry trap (363 total moths). Bars marked with different letters are significantly different. Kruskal–Wallis Test followed by a Dunn test (α = 0.05).

In field trials in Imperial Co., neither transect (*p* = 0.79) nor trap date (*p* = 0.06) were significantly different, so data from all traps were combined into a single dataset. Green Unitraps captured more moths than either the clear Unitraps (*p* = 0.01) or Scentry traps (*p* = 0.03) (Figure 1) (Kruskal–Wallis test, α *=* 0.05). Despite the numerous honeybees collecting pollen on the corn, very little bycatch was observed in traps (<3 non-target insects per trap per collection).

**Figure 2:**
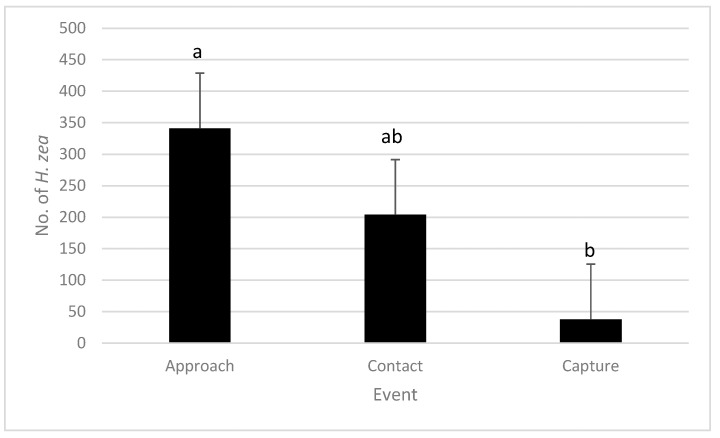
Total number of *H. zea* interacting with pheromone-baited traps. Three events were recorded: approach, contact and capture. Bars marked with different letters are significantly different. Kruskal–Wallis Test followed by a Dunn test (α = 0.05).

In field observations in Riverside, fewer *H. zea* were captured in traps than those that approached traps (Kruskal–Wallis Test, *p* < 0.05) (Figure 2). However, there was no significant difference between the number of moths approaching traps and the number of contacting traps, nor was there a difference between the number of moths contacting traps and the number captured (Figure 2).

**Figure 3:**
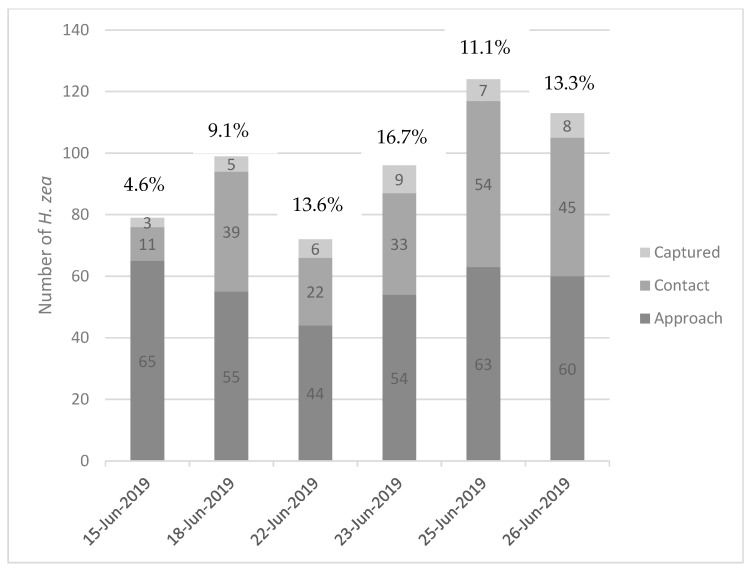
Behaviors of *H. zea* observed on nightly recordings of green Unitraps in the field. The percentages above each bar represent the trap efficiency for that night: (Number of moths captured)/(Total number of moths) × 100. Total numbers of moths exhibiting each behavior are displayed in each bar.

The efficiency of the traps can be calculated as: (Number of moths captured)/(Total number of moths) × 100. Here, the “total number of moths” indicates the number of individuals observed during the experiment regardless of the level of behavior they exhibited. This calculation was made for each night of observations (Figure 3). The minimum observed trap efficiency of green bucket traps with this lure formulation was 4.6%, while the maximum observed was 16.7%, with an average efficiency of 11.4% (±4.2%), i.e., approximately 11% of moths that approached traps were actually captured. In one instance, a moth approached a trap, disappeared into the funnel, and emerged to fly away (Video S1).

**Figure 4:**
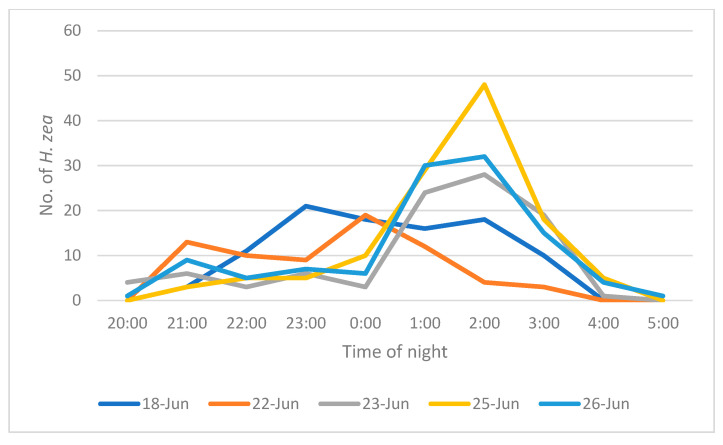
Number of *H. zea* males approaching traps during 1 h intervals throughout each night.

Males approached traps throughout the night starting from before 20:00 and ending by 05:00. The peak active period seemed to occur between midnight and 04:00 (Figure 4).

## 4. Discussion

Despite the outperformance of green Unitraps over the clear Unitrap and the Scentry trap, video observations of traps revealed that there were far more approaches than actual captures of adult moths. Although retention of captured moths was a concern, it seems that most moths that avoid capture never made it into the funnel. Thus, rather than a retention issue, the lack of captures stem from the behavior of a male *H. zea* who tended to approach the trap, contact the lure basket, and fly away, rather than descend into the funnel.

In field observations, moths approached traps throughout the night, beginning in the evening at 20:00 h, not long after sunset, and as late into the morning as 04:00 h. This apparently continual arrival at a trap throughout the night poses the question as to why more males do not approach the trap early in the night. In other words, if a moth is in the cornfield at dusk, why would it wait until 04:00 to approach the trap? One explanation is that males are flying into the field from other areas. The weekly insecticide regimen would suggest that most of the moths flew in from distant sites rather than emerging from the experimental plot. In other words, the peak activity later in the evening may be due to the time it takes moths to arrive at the field after sunset.

Alternatively, it may be that some males repetitively approached a trap. Indeed, with so many approaches and so few captures, it is possible that some of the moths observed were the same individuals visiting the trap multiple times. Possible repeat visits present a challenge when attempting to predict the actual population; neither the total captured, nor the total number of approaches can reliably estimate the true population density in a field, and other survey techniques may be required [24]. However, understanding the efficiency of traps could help growers more accurately gauge when treatments may be necessary. For example, a trap that is less efficient may signal that intervention is necessary at lower trap counts.

The possibility that moths repeatedly visited the trap also poses a challenge for interpreting the results of the present study. It could be that repeat visits exhaust males, making it more likely for them to fall into the funnel to be captured. If this is the case, then the functional efficiency of Unitraps may be higher than estimated herein.

Multiple visits by the same moths throughout the night may have implications for trap spacing in tests comparing different trap types or lure formulations. If traps are far apart, they may sample different local populations, but too close and they may interfere with each other in attracting moths [25]. If moths are not captured after their approach, they may be able to sample the plumes of several traps in a transect. In this way, traps in a study may be more akin to choice tests than independent variables. Such trap interference has been recorded for oriental fruit moth, *Grapholita molesta* (Busck) with inter-trap distances less than 30 m in an orchard, demonstrating interference between traps [26], and behavioral response of OFM at distances up to 30 m from pheromone sources have been demonstrated in field assays [27].

The deployment of traps 10 m from the edge of the corn field means that the structure of pheromone plumes emanating from the traps would not be distorted by nearby foliage. Also, there would be minimal background contrast presented by the foliage regardless of the moth’s approach direction. In contrast to our findings [28], both Hartstack wire traps and Scentry traps outperformed Unitraps in field trials of *H. zea* in North Carolina. Traps in that study were placed directly adjacent to corn or soybean plantings, possibly accounting for differences in performance. These crops would have provided a background contrast, likely to enhance the visual apparency of the traps, a factor that seems to increase capture [28]. In contrast, in an open field the plume’s structure would not be shredded by passing through foliage. Therefore, in the present study the downwind reach of the plume would be maximized [29] as would the possibility of multiple visits by the same moths.

Video recordings of insect behavior as they approach traps have been useful in investigating trap efficiency in other systems. Video recordings of palm weevils, *Rhyncophorus palmarum* (L.), demonstrated that more beetles entered Picusan^®^ traps than bucket traps, and proportionally fewer beetles escaped from Picusan^®^ traps than bucket traps [30]. These types of studies provide important understanding of insect behavior that simple trap capture data lack.

## 5. Conclusions

Unitraps, which are commonly used to monitor for *Helicoverpa zea*, are only 11% efficient when baited with a 97:3 ratio of Z11-16:Ald to Z9-16:Ald. Many moths contacted the trap and flew directly to the small, perforated basket holding the lure, but flew away without being captured. This has implications for monitoring and surveillance of *Helicoverpa* moths, as well as comparing traps within a transect because moths may be able to sample several plumes before ultimately being captured, challenging the assumption that traps are independent from one another.

## Data Availability

The original contributions presented in the study are included in the article/Supplementary Materials, further inquiries can be directed to the corresponding authors.

## Supplementary Materials

The following supporting information can be downloaded at: https://www.mdpi.com/article/10.3390/insects16050525/s1, Video S1: Male *H. zea* enters and leaves a Unitrap. Data S1: raw data gathered from the experiments.
